# Fluorophore labeling of a cell-penetrating peptide significantly alters the mode and degree of biomembrane interaction

**DOI:** 10.1038/s41598-018-24154-z

**Published:** 2018-04-20

**Authors:** Sofie Fogh Hedegaard, Mohammed Sobhi Derbas, Tania Kjellerup Lind, Marina Robertnova Kasimova, Malene Vinther Christensen, Maria Høtoft Michaelsen, Richard A. Campbell, Lene Jorgensen, Henrik Franzyk, Marité Cárdenas, Hanne Mørck Nielsen

**Affiliations:** 1 Department of Pharmacy, Faculty of Health and Medical Sciences, University of Copenhagen, Universitetsparken 2, 2100 Copenhagen, Denmark; 2 Department of Biomedical Science and Biofilms - Research Center for Biointerfaces, Faculty of Health and Society, Malmö University, Per Albin Hanssons väg 35, 214 32 Malmö, Sweden; 3 Department of Drug Design and Pharmacology, Faculty of Health and Medical Sciences, University of Copenhagen, Jagtvej 162, 2100 Copenhagen, Denmark; 4 Institut Laue-Langevin, 71 avenue des Martyrs, CS20156, 38042 Grenoble, France; 5 Present Address: Symphogen A/S, Pederstrupvej 93, 2750 Ballerup, Denmark

## Abstract

The demand for highly efficient macromolecular drugs, used in the treatment of many severe diseases, is continuously increasing. However, the hydrophilic character and large molecular size of these drugs significantly limit their ability to permeate across cellular membranes and thus impede the drugs in reaching their target sites in the body. Cell-penetrating peptides (CPP) have gained attention as promising drug excipients, since they can facilitate drug permeation across cell membranes constituting a major biological barrier. Fluorophores are frequently covalently conjugated to CPPs to improve detection, however, the ensuing change in physico-chemical properties of the CPPs may alter their biological properties. With complementary biophysical techniques, we show that the mode of biomembrane interaction may change considerably upon labeling of the CPP penetratin (PEN) with a fluorophore. Fluorophore-PEN conjugates display altered modes of membrane interaction with increased insertion into the core of model cell membranes thereby exerting membrane-thinning effects. This is in contrast to PEN, which localizes along the head groups of the lipid bilayer, without affecting the thickness of the lipid tails. Particularly high membrane disturbance is observed for the two most hydrophobic PEN conjugates; rhodamine B or 1-pyrene butyric acid, as compared to the four other tested fluorophore-PEN conjugates.

## Introduction

The interest in biopharmaceuticals, such as peptides and proteins, for the treatment of life-threatening diseases, is continuously on the rise in biomedical development. However, the relatively large molecular size and hydrophilic nature of most therapeutic peptides and proteins constitute challenging factors for achieving sufficient delivery to their target site. Their physico-chemical properties limit their ability to permeate across the cell membrane and thus reach the site of action in the body. There is an increasing need for highly efficient and non-toxic carriers to facilitate permeation of therapeutic peptides and proteins across biomembranes and to enhance the pharmacological effects of biopharmaceuticals. Cell-penetrating peptides (CPPs) seem to fulfill these criteria and have the potential to become an important tool in pharmaceutical research. CPPs are short (5–40 amino acid residues) and predominantly cationic peptides that efficiently internalize into eukaryotic cells^1^. They possess the ability to co-internalize other molecules into the cells and thus facilitate the delivery of a therapeutic cargo^2,3^. Currently, cationic CPPs are believed to interact directly with negatively charged head groups of lipids in the plasma membrane, presumably through electrostatic interactions^4–6^. In this way, CPPs increase the local peptide concentration at the membrane surface, which subsequently causes a transient destabilization of the lipid bilayer and finally leads to cell entry^7,8^. However, also hydrophobic interactions are found to be important for the CPP-membrane interaction and cellular internalization^5,9^, which specifically may be facilitated by the presence of tryptophan residues in the peptide sequence^6^.

One of the most studied CPPs is L-penetratin (PEN) derived from the homeoprotein Antennapedia^10^. Several mechanisms of internalization have been suggested for this 16-residue amphiphilic peptide. Initially, endocytosis-independent cellular internalization mechanisms were proposed for PEN, including direct translocation involving transmembrane pore formation in the lipid bilayer^11–14^. However, the majority of recent studies on the cellular uptake mechanism of PEN suggests that endocytosis is the major mechanism of uptake^8,13–15^. Regardless of the exact mechanism of cell entry for most CPPs, the plasma membrane constitutes a significant barrier, and therefore elucidation of the details in CPP-membrane interactions at the molecular level is crucial for understanding the internalization process(es) of the CPPs and their cargos^16,17^.

In order to obtain sufficient detection sensitivity when investigating the degree and mechanisms of membrane interaction, translocation propensity, and intracellular trafficking, CPPs are often labeled with fluorophores suitable for monitoring cellular uptake, e.g., by flow cytometry^5,18–20^, or for visualizing cellular distribution by confocal laser-scanning microscopy^18,19,21,22^. Although fluorescence detection technologies are highly valuable tools, recent studies have raised concerns about the often uncritical application of fluorophore-labeled short peptides, such as CPPs in mechanistic investigations^1,23^. Modification of a short, flexible, and hydrophilic peptide with a rigid, bulky, and hydrophobic moiety will unavoidably alter the physico-chemical properties of the peptide, which in turn leads to the  altered mode and/or degree of interaction with the biological membrane as previously reported^22^. To translate and utilize findings from experiments on, e.g., cellular uptake of CPPs, it is important that the effect of applying modified compounds is recognized. However, the effect of fluorophore-labeling on the interactions between short peptides and cell membranes is poorly understood. Indeed, erroneous and conflicting conclusions on the mode of membrane interaction of the peptide could arise from the use of different fluorophore moieties.

**Figure 1:**
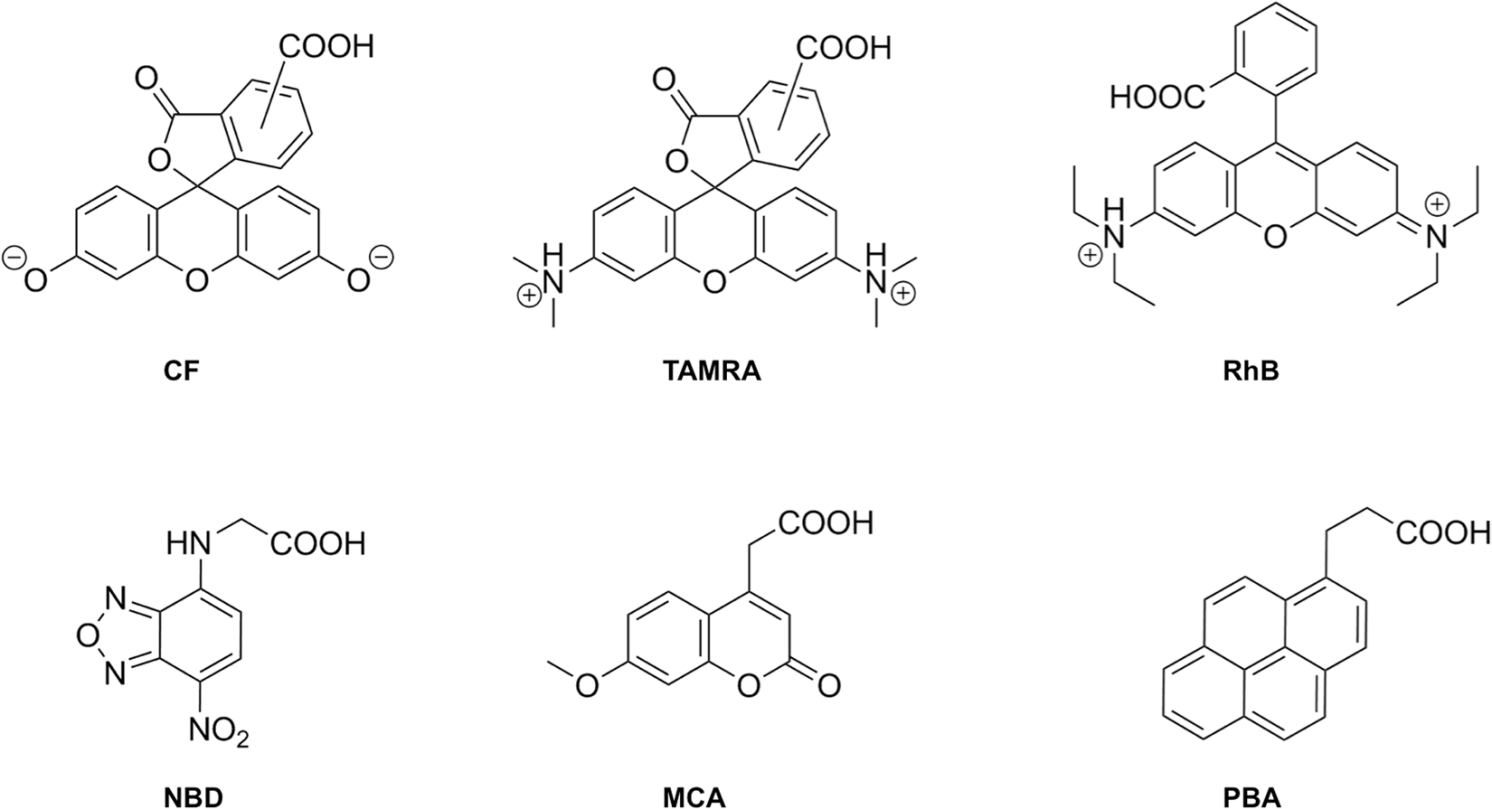
Molecular structures of the assessed fluorophores at physiological pH. **: CF:** 5(6)-carboxyfluorescein, **TAMRA:** 5(6)-carboxytetramethylrhodamine, **RhB:** N-(9-(2-carboxyphenyl)-6-(diethylamino)-3H-xanthen-3-ylidene)-N-ethylethanaminium, **NBD:** N-(7-nitro-2,1,3-benzoxadiazol-4-yl)glycine, **MCA:** (7-methoxycoumarin-4-yl)acetic and **PBA:** 1-pyrenebutyric acid.

In a recently published paper^23^, we demonstrated, with the use of several mammalian cell types, that the labeling of PEN with a series of fluorophores affected the properties of PEN in ways that notably influenced the interaction with cells; namely the cellular distribution, cytotoxicity and membrane permeability. The aim of the present work is to elucidate in detail the impact that labeling of PEN with the same fluorescent moieties (Fig. 1) has on membrane interactions. Here, we apply a series of biophysical techniques in order to obtain a fundamental understanding of the crucial first step in CPP-membrane interaction by utilizing model phospholipid-based vesicles^5,16,24^ and supported lipid bilayers (SLBs)^25–28^.

Lipid vesicles and SLBs are extensively used as model systems for studying the interactions of membrane-interacting peptides with biological membranes. The effects of a peptide on the integrity of lipid vesicles and the colloidal stability of these are assessed by using a calcein release assay and dynamic light scattering (DLS), while the enthalpy related to these interactions is measured by using isothermal titration calorimetry (ITC). The extent of peptide binding to SLBs is followed by using quartz crystal microbalance with dissipation monitoring (QCM-D). In addition, induction of peptide conformation upon binding to lipid vesicles is determined by circular dichroism (CD) spectroscopy and neutron reflectivity (NR) is applied to provide structural details on the extent of membrane insertion and distribution of the peptides.

## Materials and Methods

### Chemicals and reagents

1-Palmitoyl-2-oleoyl-*sn*-glycero-3-phosphocholine (POPC) and 1-palmitoyl-2-oleoyl-*sn*-glycero-3-[phospho-*rac*-(1-glycerol)] (sodium salt) (POPG) was purchased from Avanti Polar Lipids (Alabaster, AL, USA). Trizma base (Tris), Hanks’ balanced salt solution (HBSS), sodium azide (NaN_3_), calcium chloride dihydrate (CaCl_2_), ethylenediaminetetraacetic acid disodium salt dihydrate (EDTA), calcein, Triton™ X-100, methanol >99.9% (w/w) (MeOH), and trifluoroacetic acid (TFA) were all purchased from Sigma-Aldrich (Buchs, Switzerland). The buffer agent 4-(2-hydroxyethyl)-1-piperazineethanesulfonic acid (HEPES) was obtained from PanReac AppliChem (Darmstadt, Germany), potassium chloride (KCl) was from Merck (Copenhagen, Denmark), and ethanol 99.9% (w/w) and chloroform were obtained from Th. Geyer (Roskilde, Denmark). Medium grade sephadex-G-50 columns were purchased from GE Healthcare Life Sciences (Brøndby, Denmark). For pH adjustments of all buffer solutions, sodium hydroxide (NaOH) and hydrochloric acid (HCl) from VWR Bie & Berntsen (Søborg, Denmark) were used. Ultrapure water from a Barnstead NanoPure system (Thermo Scientific, Waltham, MA, USA) was used throughout the studies.

### Peptide synthesis, purification and labeling

L-penetratin (PEN) and N-terminally fluorophore-conjugated PEN were synthesized, purified and characterized as previously described^23^. The used peptide and conjugates were: PEN (H_2_N-RQIKIWFQNRRMKWKK-CONH_2_, Mw = 2246 Da), 5(6)-carboxyfluorescein-penetratin (PEN_CF_, Mw = 2604 Da), 5(6)-carboxy-tetramethyl-rhodamine-penetratin (PEN_TAMRA_, Mw = 2658 Da), N-(7-nitro-2,1,3-benzoxadiazol-4-yl)glycine-penetratin (PEN_NBD_, Mw = 2465 Da), 7-methoxycoumarin-4-ylacetic acid-penetratin (PEN_MCA_, Mw = 2461 Da), N-(9-(2-carboxyphenyl)-6-(diethylamino)-3H-xanthen-3-ylidene)-N-ethylethanaminium-penetratin (PEN_RhB_, Mw = 2670 Da), 1-pyrene butyric acid-penetratin (PEN_PBA_, Mw = 2515 Da).

### Preparation and characterization of lipid vesicles

For CD, ITC, and calcein release assays, lipid vesicles of the zwitterionic POPC lipid and the negatively charged POPG lipid at a composition of 80:20% (mol/mol), were prepared at room temperature by mixing appropriate amounts of lipid in chloroform stock solution and drying the lipid mixture in a round-bottomed flask by using a rotary evaporator. To remove trace amounts of chloroform, the lipid films (20–50 mg in total) were stripped three times during 3 h with 0.5 mL absolute ethanol and dried overnight on the rotary evaporator. The POPC:POPG lipid films were rehydrated in buffer. To obtain lipid vesicles for the CD studies, the rehydration buffer was Tris buffer (10 mM, pH 7.4), and for the ITC studies HEPES buffer (10 mM, pH 7.4) was used. To obtain calcein-loaded lipid vesicles for the calcein release assay, isotonic HEPES buffer (10 mM HEPES, 150 mM KCl, 1 mM NaN_3_, 30 µM CaCl_2_, 10 µM EDTA in water, pH 7.4) containing 70 mM calcein was used. Following rehydration, the lipid dispersion was sonicated for 5 min in an ultrasonic bath (model 2510, Branson, Danbury, USA) to avoid aggregates, vortexed thoroughly every 10 min during a 60 min period, and then left to anneal for 1 h before extrusion. To obtain homogenous lipid vesicles, extrusion was done twice through a double layer of 200 nm Nuclepore polycarbonate membrane filters (Whatman, Florham Park, NJ, USA), followed by nine extrusion rounds through double layers of 100 nm Nuclepore polycarbonate filter (Whatman, Florham Park, NJ, USA). Upon complete extrusion, the calcein-loaded lipid vesicle dispersions were purified three times using a Sephadex G-50 column (GE Healthcare, Little Chalfont, UK).

For QCM-D monitoring, SLBs were formed on silicon oxide crystals, 50 nm (Q-Sense, Västra Frölunda, Sweden) and for NR experiments on silicon(111) surfaces by *in-situ* vesicle fusion as described previously^24,25^. Lipid vesicles (POPC:POPG, 80:20% (mol/mol)) for this purpose were prepared at room temperature by mixing lipid stock solutions in chloroform and drying the lipid mixture under a stream of nitrogen in glass vials. The lipid films (1 mg) were then left in vacuum for 3 h to remove any trace amounts of chloroform, and kept at −20 °C until use. On the day of the experiment, a lipid film was hydrated with ultrapure water, vortexed thoroughly every 10 min during a 60 min period, and then tip-sonicated a few minutes using a Branson sonifier (model 250/450, Danbury, CT, USA) until the lipid vesicle dispersion appeared clear. Immediately before the  formation of the SLB by vesicle fusion, the lipid vesicle dispersion was diluted to a final concentration of 0.1 mg lipid/mL in 2 mM CaCl_2_ in ultrapure water.

### Lipid quantification of calcein-loaded lipid vesicles

After purification, the lipid concentration in calcein-loaded lipid vesicle dispersions was determined by reversed-phase high-pressure liquid chromatography (RP-HPLC) with an Agilent 1260 Infinity system (Agilent Technologies, Waldbronn, Germany) equipped with a temperature-controlled auto sampler, column oven, binary pump, and a mobile phase degasser. The system was coupled to an evaporative light-scattering detector (ELSD), operating at a gas flow of 1.5 L/min, a nebulizer temperature of 50 °C, and an evaporator temperature of 90 °C. The lipids were separated by a Waters SunFire C8 column (150 × 2.1 mm, 3.5 µm particle size, 100 Å pore size; Waters, Milford, MA, USA) at 50 °C. Eluents A (95/5/0.1 (v/v/v) water/MeOH/TFA) and B (95/5/0.1 (v/v/v) MeOH/water/TFA) were employed with a flow rate of 0.3 mL/min using a linear gradient of 75 → 100% B for 15 min, followed by a 10 min plateau at 100% B. Limit of detection (LOD) and limit of quantification (LOQ) were found to be 8.4 and 25.1 µmol for POPC, respectively, and 18.5 and 56.2 µmol for POPG, respectively, in the linear range of 100–2000 µmol.

### Circular dichroism spectroscopy

The peptide was dissolved in Tris buffer and mixed with freshly prepared lipid vesicles in a lipid-to-peptide molar ratio of 100:1, with a peptide concentration of 20 µM and a total lipid concentration of 2 mM in Tris buffer. Tris buffer was used due to its low absorbance above 190 nm^26^. CD spectra were obtained with a Chirascan Plus Circular Dichroism spectrometer (Applied Photophysics, Leatherhead, UK) using a 0.1 cm quartz cuvette. The spectra were acquired in the 190–280 nm range in steps of 0.5 nm at 25 °C. Each spectrum represents an average of 4 scans. Applied Photophysics Pro-Data Chirascan software (Chirascan Spectrometer Control Panel Application Version 4.4.2.0) was used for data processing. The experiment was carried out once on the same vesicle batch.

### Isothermal titration calorimetry

Prior to ITC analysis, the lipid vesicle dispersion was dialyzed against HEPES buffer by using a 3500 MWCO Slide-A-Lyzer dialysis cassette (Thermo Scientific, Rockford, IL, USA) in a lipid-to-buffer volume ratio of 1:200 for 2 h at room temperature. Buffer and peptide solutions were degassed under vacuum prior to use to eliminate air bubbles. The calorimeter cell was filled with 177 µL peptide solution, and the titrations were performed by injecting 1.96 µL aliquots of POPC:POPG vesicles (80:20 % (mol/mol), 20 mM total lipid concentration) into the cell at time intervals of 300 s. The heat of dilution was determined by titrating lipid vesicles into HEPES buffer. All measurements were performed with a low volume Nano-ITC 2G (TA Instruments, New Castle, DE, USA) at a stirring rate of 300 rpm. Baseline correction and peak integration were conducted by using the NanoAnalyze software (TA instruments, New Castle, DE, USA). Data analysis was conducted in Excel, version 14.6.7 (Microsoft, Houston, TX, USA). Heat associated with the first injection (0.48 µL) was not included in the data analysis. All experiments were carried out at 37 °C in duplicates or triplicates on the same lipid vesicle batch. The lipid vesicles were used within three weeks of preparation, and dynamic light scattering (DLS) size measurements were applied to check the colloidal stability during the period of use.

### Calcein release assay

Calcein-loaded lipid vesicles (100 µM lipid) were diluted four times with the peptide solutions to reach a final lipid concentration of 25 µM. The peptide was dissolved and diluted in hHBSS buffer (10 mM HEPES in HBSS pH 7.4) to give appropriate concentrations. Immediately before starting the experiment, the peptide solutions were mixed with the lipid vesicle dispersion to a final volume of 180 µL in wells of NUNC™ black 96-well plates with optical flat bottom (Thermo Fisher Scientific, Waltham, MA, USA). The initial calcein fluorescence (F_0_) signal was measured at λ_ex_ = 485 nm and λ_em_ = 520 nm, and the fluorescence (F) was recorded every 10 s for 40 min at 37 °C. At the end of the experiment, the maximum fluorescence (F_t_) signal was measured after the addition of 20 µL 2% (v/v) Triton™ X-100 to all wells to induce complete calcein release. A POLARstar Optima plate reader (GMB Labtech, Offenburg, Germany) was applied to all measurements and equation (1) was used to calculate the degree of calcein release.

1 $$Calcein\,leakage\,( \% )=[\frac{(F-{F}_{0)}}{({F}_{t}-{F}_{0})}]\cdot 100\, \% $$

The fluorescence signal from the fluorophore-labeled peptide conjugates dissolved in buffer in the same concentrations was measured in parallel to the conducted experiments with lipid vesicles. This was performed to assure that the fluorescence signal from the labeling moieties did not interfere with the signal from calcein. Any measured background fluorescence was subtracted from the data. All experiments were conducted in triplicates on the same batch of calcein-loaded lipid vesicles within three weeks of preparation.

### Quartz crystal microbalance with dissipation monitoring

The flow cells of a Q-SENSE E4 system (Q-Sense, Västra Frölunda, Sweden) were connected to a peristaltic pump (Ismatec IPC, Glattbrugg, Switzerland) employing a flow rate of 100 μL/min throughout all experiments. The peptide, dissolved in HEPES buffer at a concentration of 5 µM, was introduced with  the flow to the preformed SLB for 8 min. After that, the flow was stopped for 15 min followed by buffer rinsing conducted for minimum 20 min. Prior to experiments, the fundamental frequency (5 MHz) and five overtones (3^rd^, 5^th^. 7^th^, 9^th^ and 11^th^) were found and recorded in ultrapure water. To obtain a stable baseline, the signals were recorded overnight prior to the experiments. All experiments were carried out at 25 °C and repeated at least three times.

### Neutron reflection

Specular neutron reflection measurements were performed at the Institut Laue-Langevin (Grenoble, France) using the time-of-flight reflectometer FIGARO^27^. The clean silicon crystals were characterized in H_2_O and D_2_O. For the construction of the SLB, the lipid vesicle solution was allowed to incubate on the silicon crystal for minimum 15 min before rinsing with H_2_O-based isotopic contrast HEPES buffer to remove excess lipids. The SLBs were characterized by using three isotopic buffer contrasts; H_2_O, D_2_O, and cm3 (contrast matched to the scattering length density (SLD) equal to 3 × 10^−6^ Å^−2^). Using a syringe pump, 30 mL of 5 µM peptide in H_2_O HEPES buffer was subsequently introduced to the SLB with a flow rate of 3 mL/min. The sample cell was rinsed with HEPES buffer of one of the three contrasts (H_2_O, cm3, and D_2_O) prior to full characterization in each of the contrasts. The specular reflection was measured as a function of the scattering vector (Q) according to equation (2),

2 $$Q=\frac{4\pi }{\lambda }\,\sin (\theta )$$

where λ is the wavelength of the incident beam and θ is the angle between the incoming beam and the reflecting surface. Measurements were made using neutrons of λ = 2–20 Å with θ = 0.8° and 3.2°. Data were normalized with respect to a measurement of the silicon crystal in D_2_O. The SLD, thickness (d), solvent penetration (φ), and interfacial roughness between the layers (σ) were the parameters used to characterize the interfaces. The experiments were carried out at a temperature of 25 °C, controlled via an external water bath loop. All NR profiles were analyzed using the Motofit package as previously described^28^. The clean crystal was fitted to a two-layer model (Si-SiO_2_), whereas the best fit for the bilayer was obtained with a three-layer model consisting of head groups-lipid tails-head groups. Modeling the SLB after interaction with peptide required additional layers, as described in the results section.

## Results

The molecular structures of the fluorophore labels investigated in this study are presented in Fig. 1. These fluorophores were chosen because all of them are commonly used for detection by using various fluorescence-based techniques^5,19,29,30^. In order to investigate how the fluorophore moieties affect the interaction of PEN with a lipid bilayer membrane, several biophysical methods were employed providing information on; (i) the conformation of the peptide conjugates in the presence of lipid vesicles, (ii) the colloidal stability of lipid vesicles, (iii) the permeability and structure of the membrane, and (iv) the thermodynamics of the binding process.

### Effect on peptide structure and folding propensity

**Figure 2:**
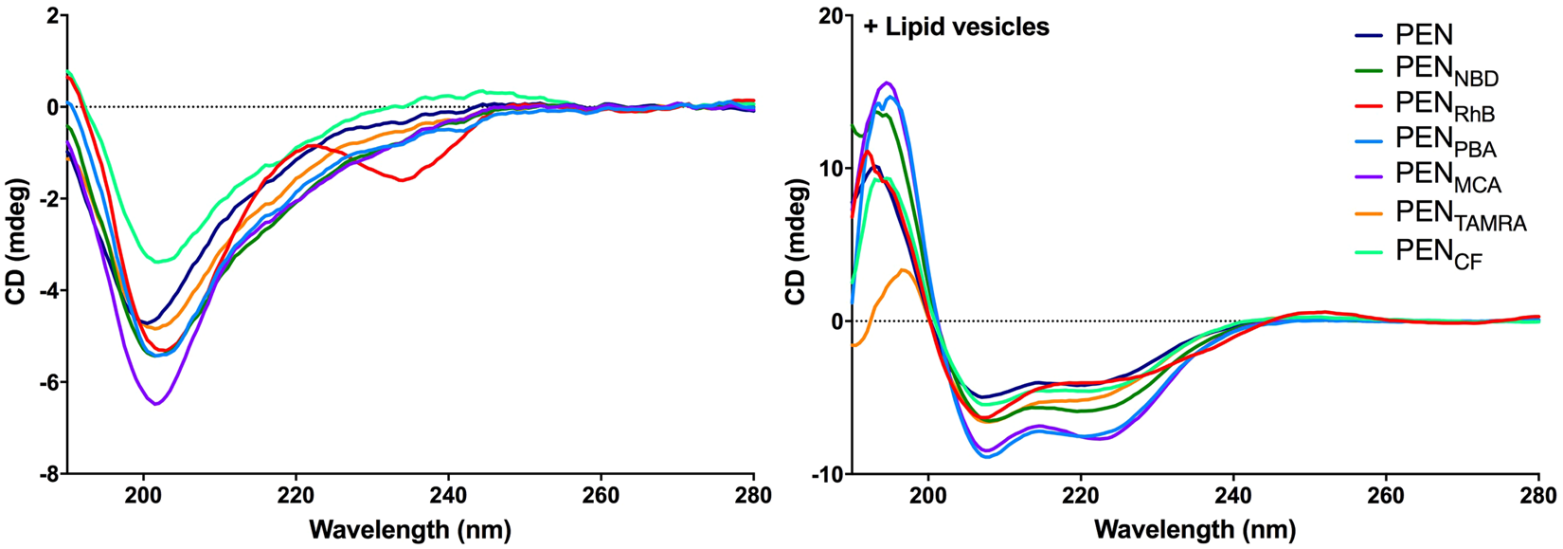
Secondary structure of fluorophore-labeled PEN in the presence of lipid bilayers as measured by circular dichroism at 25 °C. Fluorophore-PEN conjugates (20 µM) in the absence (left) or in the presence (right) of POPC:POPG (80:20% (mol/mol)) lipid vesicles with a total lipid concentration of 2 mM.

CD was applied to determine the folding propensity of the  fluorophore-labeled PEN in the  buffer and in the presence of lipid vesicles (Fig. 2). A conformational change from the  random coil in the  buffer to α-helical structures upon lipid vesicle addition was observed for all the peptide conjugates, as detected by the characteristic patterns of the CD signal, which for α-helices are recognized by the two minima in the CD signal at 208 nm and 222 nm^31^. The fluorophores affected the conformation of PEN in solution (e.g. PEN_RhB_) as well as the degree of α-helix formation upon membrane interaction, e.g., the intensity at 222 nm was almost twice for PEN_PBA_ and PEN_MCA_ in the presence of lipid that of the unmodified PEN. However, none of the N-terminally conjugated fluorophores induced any decrease in the α-helical folding propensity of PEN (e.g., a lower CD signal was observed), and thus, did not compromise the ability of PEN to adopt a secondary structure upon interaction with the lipid bilayer.

### Effect on membrane permeability

**Figure 3:**
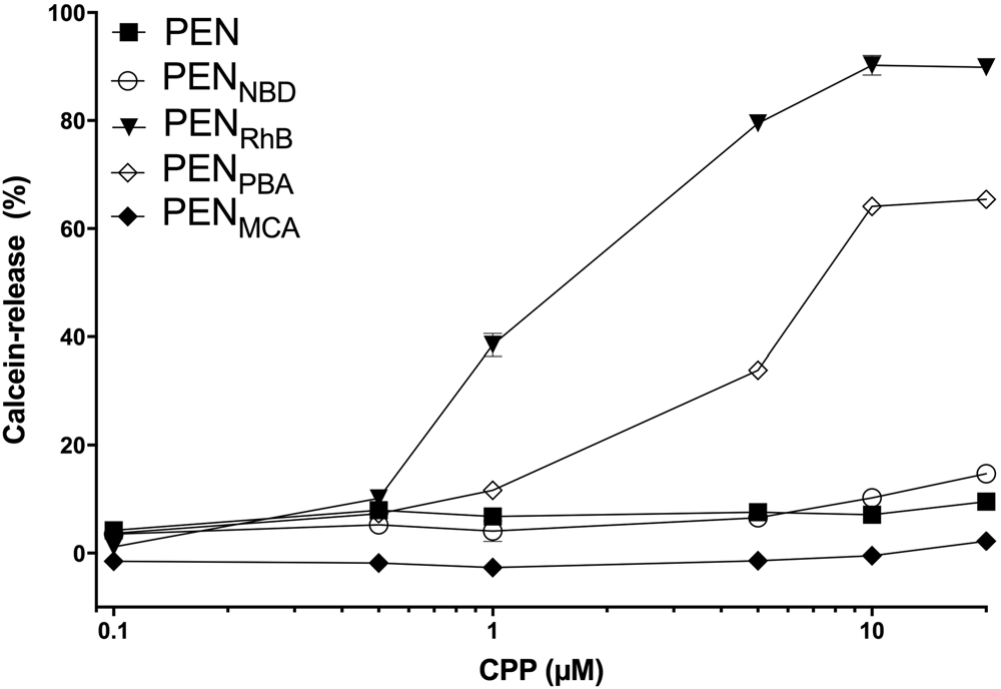
Calcein release from POPC:POPG (80:20 % (mol/mol)) lipid vesicles (25 µM) incubated for 40 min with fluorophore-PEN conjugates at different concentrations at 37 °C. Mean ± SD, n = 3. Errors are included, but are smaller than the size of the symbols indicating data points.

The membrane permeability inducing capacity of the fluorophore-PEN conjugates was investigated by monitoring the release of encapsulated calcein from phospholipid vesicles. The calcein dye leakage method is a well-established approach to study the effect of membrane-interacting molecules on lipid membranes^12,32,33^. In the absence of a CPP, low background fluorescence signals were observed throughout the experimental period due to the self-quenching of calcein at the concentrations inside the lipid vesicles. No leakage from lipid vesicles occurred under these conditions. The calcein release from POPC:POPG vesicles after 40 min exposure to PEN and each of the labeled conjugates at a peptide-to-lipid molar ratio range of 1.25–250 was calculated according to equation (1), and results are depicted in Fig. 3. It was not possible to obtain reliable data from PEN_CF_ and PEN_TAMRA_ in this assay: the emission wavelength of PEN_CF_ (λ_em_ = 520 nm) is identical to the emission wavelength of calcein (λ_em_ = 520 nm), and thus it is not possible to differentiate the compound fluorescence from the calcein signal. Moreover, the absorption spectrum of PEN_TAMRA_ is partly overlapping with the emission spectrum of calcein, which infers that light emitted by calcein at λ_em_ = 520 nm is partly absorbed by PEN_TAMRA_. The results in a reduction in the fluorescence signal obtained from calcein, thereby yielding apparent reduced release and risk of false negative results.

It is evident from Fig. 3 that the extent of membrane perturbation varied depending on the tested fluorophore conjugate. A significantly higher calcein release was observed for two of the conjugates: PEN_RhB_ and PEN_PBA_. For PEN_RhB_, it was observed that the membrane leakage corresponded to almost 40% of the encapsulated calcein at conjugate concentrations as low as 1 µM, while the calcein release reached a plateau at around 90% at concentrations above 5 µM. Moreover, at concentrations above 1 µM, PEN_RhB_ induced a burst release of calcein corresponding to more than 95% of the maximum amount of calcein released within the first minute of peptide-vesicle exposure. A plateau of maximum calcein release for PEN_PBA_ was reached at approximately 65%, but the time to reach 95% of maximum released calcein was around 15 min for the two highest PEN_PBA_ concentrations tested. Less than 10% calcein was released upon exposure to non-labeled PEN even at the highest tested concentration of 20 µM, which was in accordance with previous results^5,34,35^. Exposure to PEN_NBD_ resulted in a slightly higher release of approximately 15% at 20 µM, whereas PEN_MCA_ did not affect the lipid membrane to a degree that induced significant calcein release in any of the tested concentrations.

### Thermodynamics of membrane interaction

**Figure 4:**
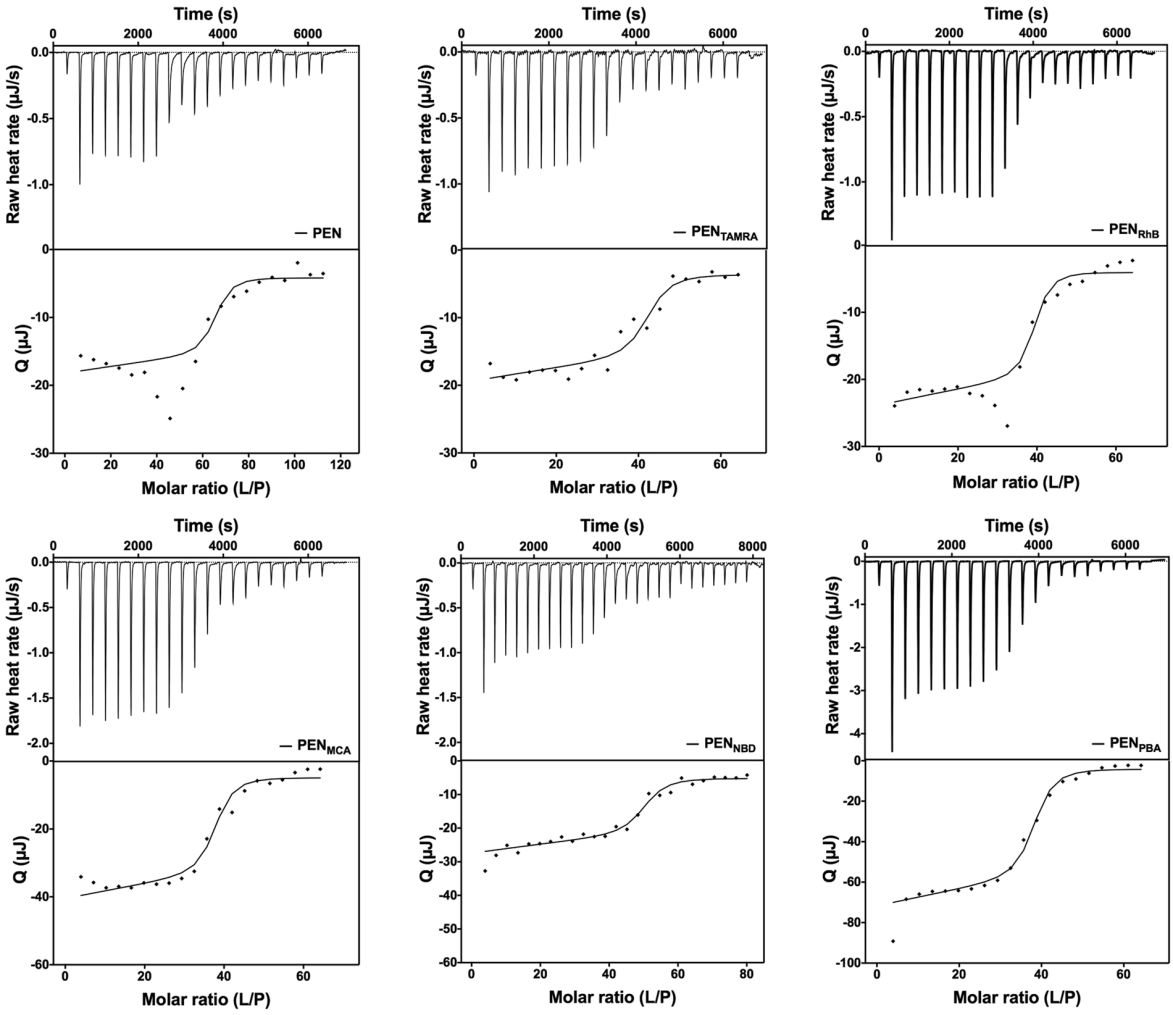
Representative titration calorimetric curves from ITC experiments conducted at 37 °C. Top graphs show the injection peaks, with each peak corresponding to the injection of 1.96 µL (except that 0.48 µL was used for the first peak) of 20 mM POPC:POPG (80:20 % (mol/mol)) lipid vesicles into 177 µL peptide in aqueous medium, pH 7.4 (70 µM for fluorophore-labeled PEN and 40 µM for PEN). Bottom graphs show the corresponding integrated injection heats as a function of lipid-to-peptide (L/P) molar ratio. The solid lines are the best fits to the data.

The thermodynamics of the interaction of PEN and fluorophore-PEN conjugates with anionic phospholipid vesicles were investigated by ITC with an  incremental injection of lipid vesicles into an aqueous medium containing the peptide. In all of the experiments, exothermic heat of reaction was observed as indicated in the representative thermograms obtained at 37 °C (Fig. 4). At the initial stage, when the lipid-to-peptide molar ratio is low, the binding capacity of the lipid is fully saturated. At this stage of the titration, several processes are taking place: (i) peptide binding to the membrane which is accompanied by structural changes of the peptide, and (ii) rearrangement of the lipids in the bilayer induced by the peptide penetration into the surface layer of the lipid vesicles. The apparent injection heat is, therefore, a sum of several processes. Upon incremental addition of lipid, the peptide conjugates were redistributed between the lipid vesicles: peptides dissociated from saturated vesicles and bound to those newly added. A decrease in the magnitude of the titration peaks was observed during the consecutive injections, subsequently leveling out to a final plateau. Further addition of lipid vesicles did not result in additional peptide binding. At this stage, no free peptide conjugates were available, and only the heat of dilution was observed.

The thermograms for PEN and PEN_RhB_ reveal a titration shape with data points deviating from the classical sigmoidal curve as observed for the other conjugates. This deviation is visible after the initial injections giving a plateau in the differential reaction heat, where the absolute value of Q increases notably prior to decreasing to values close to zero, representing only the heat of dilution. When zooming in at the baseline, it is evident that the peaks broadened in this area for especially these two peptide-conjugates. These observations were reproduced in repeated experiments. The presented isotherms were fitted to the equilibrium model describing one set of identical binding sites, and the results are summarized in Table 1. Data from the binding studies with PEN_CF_ are not included, as the recorded thermograms were not reproducible, in contrast to thermograms obtained with the other compounds (see discussion section).

**Table 1:** Thermodynamic parameters measured at 37 °C obtained from fitting the ITC data to “one-set-of-identical-binding-sites” model.

CPP	ΔH (kJ/mol)	Binding stoichiometry (n)
PEN	−0.34, −0.33	65, 63
PEN_**RhB**_	−0.46, −0.46	36, 38
PEN_**TAMRA**_	−0.39, −0.37	41, 41
PEN_PBA_^*^	−1.50 ± 0.08	38 ± 1
PEN_NBD_^*^	−0.64 ± 0.07	48 ± 5
PEN_**MCA**_	−0.83, −0.87	37, 38

*Triplicate measurements listed as mean ± standard deviation. All other compounds were measured in duplicate, and listed as values for single measurements.

PEN labeled with TAMRA, PBA, NBD, and MCA could all successfully be fitted to the “one-set-of-identical-binding-sites” model in contrast to the non-labeled PEN and RhB-PEN conjugate. In the latter cases, the data points at the beginning and the end of titrations were fitted, whereas two to three data points in the middle were excluded in the fit.

### Overall binding interaction at SLBs

**Figure 5:**
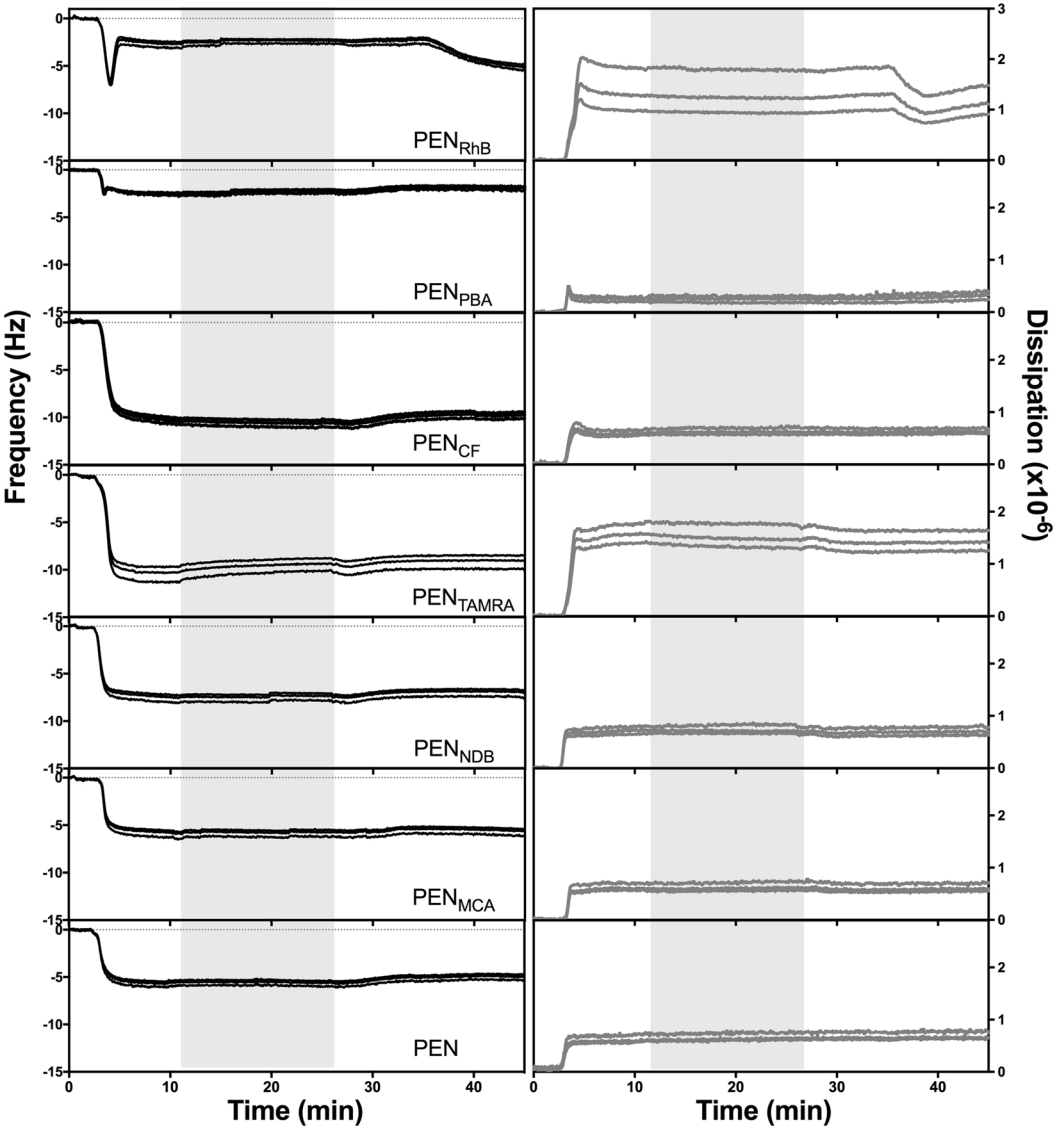
Change in frequency (left) and dissipation (right). For each of the three overtones for the conjugates; 5^th^, 7^th^ and 9^th^ are shown from representative experiments (n > 3). Supported lipid bilayers of POPC:POPG (80:20 % (mol/mol)) were formed on SiO_2_ by vesicle fusion (Fig. S1), prior to introduction of fluorophore-labeled CPP at a concentration of 5 µM at t = 2 min with 100 µL/min flow for 10 min. After 15 min of exposure (grey area) the SLBs were rinsed with buffer. The data was offset to 0 after SLB formation.

QCM-D allows for real time *in situ* studies of peptide interaction with an SLB. Simultaneous measurements of the changes in frequency and dissipation (Δf and ΔD, respectively) can provide valuable information about the structural properties of SLBs interacting with CPPs. SLBs were formed by vesicle fusion and representative traces showing the formation are given in Supplementary Fig. S1, in agreement with previous results^36,37^. Immediate changes in QCM-D signals (frequency and dissipation) occurred for PEN and all the labeled conjugates upon exposure of the peptide to the SLB (Fig. 5). The instant decrease in the frequency traces indicates a fast adsorption process of the positively charged peptide to the net negatively charged SLB. Equilibrium conditions were reached within a few minutes of a  continuous flow of the CPP. In a few cases (PEN_RhB_ and PEN_PBA_), a minimum in Δf and a maximum in ΔD were observed prior to stabilization. This suggests that a major reorganization of the lipid bilayer structure took place the latter two cases^38,39^. After a 15-min period of equilibration without flow, rinsing with buffer for 20 min resulted in no major immediate change in either Δf or ΔD signals for PEN and most of the studied PEN conjugates. This implies a strong and irreversible binding of PEN and these conjugates to the lipid membrane and/or SLB restructuring. For PEN_RhB_, however, slight decreases in both dissipation and frequency were observed after 10 min of flow. Moreover, the spreading in the dissipation overtones for PEN_RhB_ indicates that this conjugate induced an SLB that was softer than the original SLB. Similar results were observed for PEN_TAMRA_. Together the overtone spreading and instability upon rinsing with buffer suggest a  substantial detrimental effect of these two conjugates on the structure of the SLB.

### Impact of interaction on the structure of the supported lipid bilayer

QCM-D is a reasonably simple technique for fast assessment of whether the  interaction between the peptide and the membrane takes place. However, QCM-D lacks the ability to provide more detailed structural information. In contrast, NR is sensitive to isotopic composition and is a well-established technique to study SLBs^40,41^. It is a suitable method for in-depth structural investigations with a resolution down to a few Ångstrom (Å), and thus it is ideal for studying the potential integration of the conjugates into the distinct buried interfaces of a lipid bilayer. In NR, the intensity of the specularly reflected neutrons is measured as a function of the scattering vector, Q. The reflectivity profiles provide information about the scattering length density (SLD) of the layers in the direction perpendicular to the surface, and thereby information on the isotopic composition.

**Figure 6:**
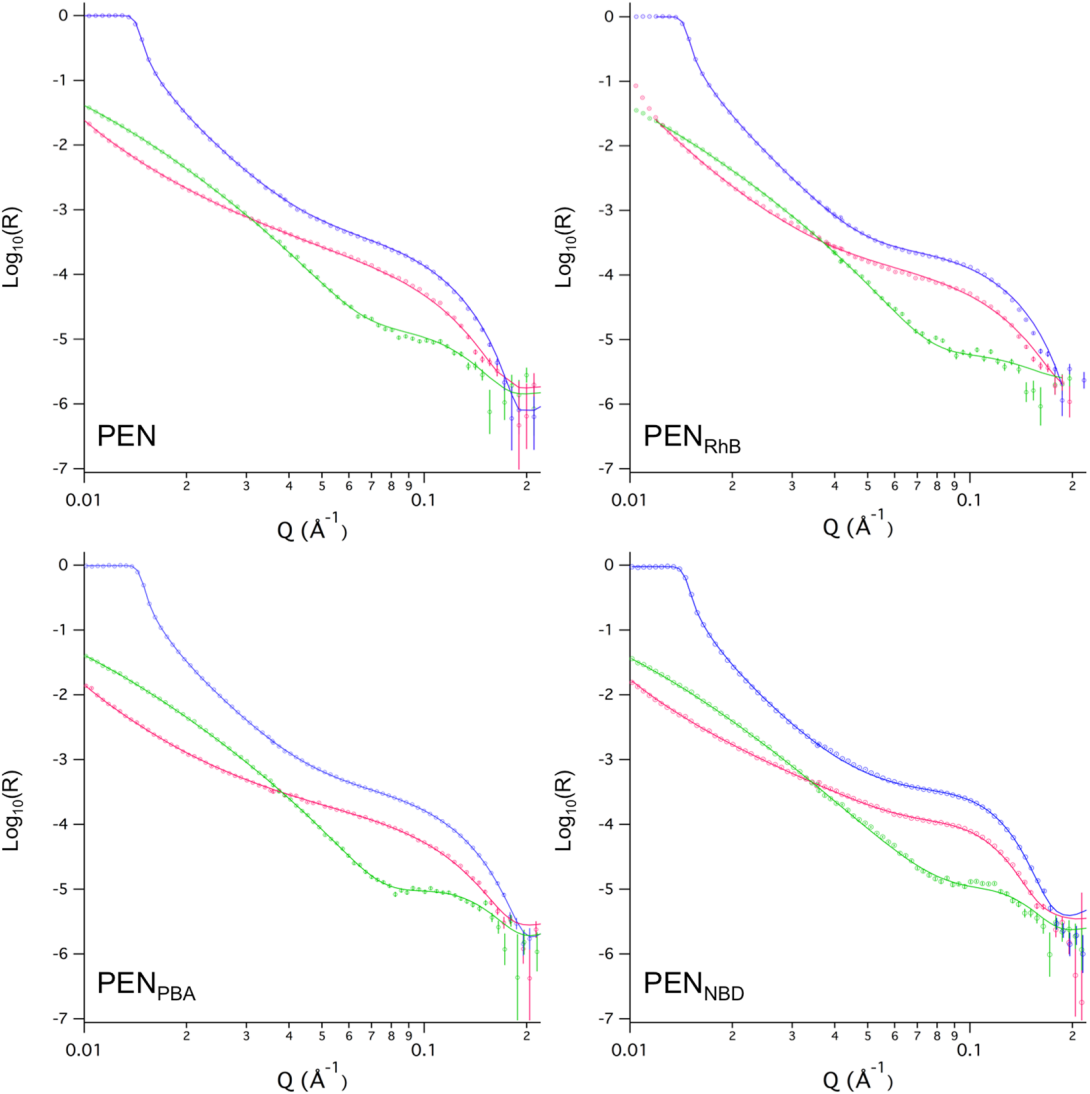
Neutron reflectivity profiles (symbols) and best fits (lines) for a POPC:POPG (80:20 % (mol/mol)) SLB exposed to a CPP in a concentration of 5 µM in HEPES buffer, pH 7.4. The SLBs were measured in three isotopic contrasts; blue: D_2_O, pink: cm3, and green: H_2_O. The parameters used in these fits are given in Supplementary Tables S1–S4.

Before investigating the effect of the fluorophore-PEN conjugates on the SLB structure, the SLB was characterized in three different isotopic buffer contrasts. The results can be found as Supplementary Fig. S2, where the symbols represent data points, and the solid lines are the best fits to a three-layer model in which head groups and tails are clearly separated (Fig. S3). On average, the head group layers were 7.5 Å, tail layers were 29.5 Å, and the mean molecular areas of the phospholipids were 65 Å^2^. These values are in agreement with previous results for SLBs of similar composition^37^. The NR profiles for the CPP-SLBs measured in three different isotopic buffer contrasts are presented in Fig. 6 and Supplementary Fig. S4. The solid lines in Fig. 6 represent the best fits to the data using a model that depends on the conjugate (see section below). The obtained fitting parameters are listed in Tables S1–S4 in the supplementary information, and SLD values and molecular volumes used in the fitting procedure are listed in Table 2. Apart from PEN itself, reflectivity profiles from the fluorophore-labeled peptide conjugates PEN_RhB_, PEN_PBA_, PEN_NBD_, PEN_CF_, and PEN_TAMRA_ were measured after interaction with the SLB (Fig. 6 and Supplementary Fig. S4).

Native PEN did not induce a major change in the SLB structure, and thus the same three-layer model was used for PEN-exposed SLB as for the SLB bilayer. However, small adjustments of the parameters used to fit these three layers were necessary. In particular, the thickness and water content of the head group region increased slightly, while 10% of the added amount of peptide was localized in the hydrophobic lipid tail region. The native PEN most likely covered the defects in the SLB as the original SLB had a dry coverage of 88% (12% defects) and therefore no actual incorporation of the  peptide into the lipid core of the bilayer took place. Moreover, upon modeling the data, the SLD value of hydrogenated PEN was allowed to vary within the theoretical range of SLD values for PEN (Table 2), to accommodate for hydrogen/deuterium exchange. This variation supports the assumption that PEN is not embedded in the lipid core, but rather is filling the defects of the SLB. Thus, PEN was predominantly associated with the head groups of the lipid bilayer, which leads to head group expansion (thicker and more solvated headgroup). Similar conclusions were drawn from an NR study on the interaction of PEN with a zwitterionic SLB in the gel phase^42^.

**Table 2:** Scattering length densities (SLD) and molecular volumes used in the fitting of NR data.

	×10^6^/Å^−2^	Å^3^
Peptide (H_2_O)	1.68*	2834
Peptide (cm^3^)	2.62*	—
Peptide (D_2_O)	3.55*	—
Head groups	2	316**
Lipid tails	−0.3	930**

*Assuming 100% exchange of labile hydrogen atoms. Ref: *Biomolecular Scattering Length Density Calculator*, http://psldc.isis.rl.ac.uk/Psldc/index.html^56^.

**POPC:POPG (80:20% (mol/mol))^57,58^.

For all the fluorophore-labeled peptide conjugates, one or more layers had to be introduced in the fitting procedure to properly model the data. These layers were placed on top of the lipid bilayer and facing the bulk aqueous phase. For PEN_RhB_, an extra peptide layer with a thickness of ~4 Å and a coverage of 16% was introduced between the underlying surface and the inner head group, with a peptide coverage of 16%. The best parameters to the fitted model state that 15% of the  peptide was co-localized within the region of the lipid tails. Given that the original SLB had a fitted surface coverage of 94% (only around 6% defects), at least 9% peptide could potentially be incorporated into the hydrophobic core. Peptide incorporation rather than filling defects is supported by the fact that no change in the SLD due to hydrogen/deuterium exchange in the peptide was necessary to fit the data. The change in thickness and increase of solvent in the head groups suggest that PEN_RhB_ is also associated with the head group areas as observed for PEN. However, a large decrease of 5 Å in thickness of the lipid tail region was observed.

A four-layer model could also be used to fit the data obtained with PEN_PBA_, suggesting it to have a similar effect on the SLB structure as observed for PEN_RhB_. A 4-Å thick peptide layer of 31% coverage existed between the surface and the inner head group. About 20% peptide was incorporated into the lipid tail region and, given that the coverage of the original SLB fitted to 100% and no hydrogen/deuterium exchange in the peptide, lipids must have been removed in order to make space available for the peptide immersion. In line with the results from PEN_RhB_, the  addition of PEN_PBA_ to the SLB also led to a decreased thickness of the lipid tail region (∼3 Å), whilst both thickness and solvation changed in the head groups. The latter clearly suggests peptide association to the head groups. Finally, a constant number of lipid molecules along the three structural layers of a SLB were not conserved for these fits, mainly due to peptide incorporation.

A more complex model with several distinct layers had to be applied to fit the NR data obtained from the SLB interacting with PEN_NBD_. The best fit obtained to the data suggests a complete reorganization of the SLB, with a mixed PEN_NBD_/lipid layer formed on top of the SLB. The remaining SLB was thinner in its lipid core and outer head group region, yielding a lower mean molecular area than the original SLB. Instead, the inner head group region was thicker, which could indicate some association of peptide with this layer (the sensitivity limit for the SLD in this layer is estimated to be at ~5% peptide incorporation). However, PEN_NBD_ was mainly localized 29 Å from the SLB in a lipid/peptide layer with a thickness of 19 Å and with 11% coverage. This implies that lipid molecules were removed from the bilayer during the interaction process, and mixed lipid/peptide structures were formed and stayed attached to the remaining SLB, most likely as a result of the binding to loosely bound peptide (the sensitivity limit for incorporation of the  peptide in this layer is ∼1%). The overall thickness of the bilayer decreased only slightly, but the mixed lipid/peptide layer had a completely different structure than that of a lipid bilayer.

In comparison to the reflectivity profiles described for PEN, PEN_RhB_, PEN_PBA_, or PEN_NBD_, the profiles generated from SLB interaction with PEN_CF_ or PEN_TAMRA_ show a more pronounced fringe in the D_2_O and cm3 contrasts at a Q-value of approximately 0.1 Å^−1^ (Supplementary Fig. S4). Similar to the experiment with PEN_NDB_, this suggests significant effects of the peptide conjugates on the structural properties of the lipid bilayer. Specific models could, however, not be obtained for studies of PEN labeled with the fluorophores CF and TAMRA due to the high structural complexity of the interfacial structure.

## Discussion

### Multiple enthalpy-driven reactions contribute to peptide-membrane binding

For most peptides, both electrostatic and hydrophobic interactions are involved in their binding to a lipid membrane^43^. Conjugation of a fluorophore to PEN might well affect the peptide-membrane interaction in several ways, e.g., by modulating the relative contributions of these two interaction forces. The attachment of a hydrophobic moiety to PEN is expected to significantly affect the contribution of hydrophobic interactions during the membrane association step as well as in a subsequent potential translocation event. The initial membrane association of membrane-interacting peptides is believed to be enthalpy-driven, facilitated by the transition of the peptide into a folded ordered state with α-helical and/or β-sheet conformation^44^. This conformational change stabilizes the peptide-membrane interaction, which is believed to be important for the subsequent translocation across the lipid membrane^45^. Thus, restriction or modulation of the ability to adopt secondary structures could potentially change the mode of membrane interaction of PEN, and thereby the efficacy of membrane penetration^46^. However, the conjugation of hydrophobic fluorophores did not limit the ability to adopt a defined secondary structure upon membrane interaction (Fig. 2) at the molar range tested in this study which corresponded to concentrations used in cell experiments^47,48^.

Our data partially confirm the above considerations, since binding of PEN_MCA_ and PEN_PBA_ to the membrane results in a significantly higher enthalpy (Fig. 4, Table 1) and a  higher degree of formation of secondary structure than for the other conjugates (Fig. 2). However, the consistently low binding enthalpy does not amount to the previously estimated −0.7 kcal/mol/residue^49^, and thus suggesting that other enthalpy-driven interactions contribute to this process.

### Peptide-induced vesicle assembly reflects molecular rearrangements

The majority of the ITC titration profiles obtained when titrating lipid vesicles into PEN and fluorophore-labeled PEN derivatives display a classic sigmoidal shape, indicative of a binding reaction of the “one-set-of-identical-binding-sites” type (Fig. 4). However, in the cases of PEN and PEN_RhB_, the sigmoidal curve is interrupted by a series of peaks with larger negative heats than the rest of the titration. This burst in binding enthalpy occurs in the same position of the titration curve, where the peptide-lipid ratio is close to the inflection point. This suggests the existence of an additional process for PEN and PEN_RhB_, e.g., peptide penetration, lipid restructuring or a combination thereof. Also, the individual peaks located in the middle of titrations become broader, indicating the existence of a slow process, such as lipid rearrangement as an example.

DLS experiments (See Supplementary Fig. S5), designed to mimic ITC titrations by using the same lipid-to-peptide ratios, show that all the investigated peptide conjugates induce vesicle assembly in the molar ratios around the inflection point of the ITC titration curves. Previous results have indeed shown that PEN can induce assembly of lipid vesicles at certain lipid-to-peptide ratios^34,50,51^. This assembly was reversible, as the vesicles recovered their original size at high lipid-to-peptide ratios, suggesting the absence of lipid fusion.

In contrast to the other investigated labeled peptide conjugates, assembly of vesicles is induced at low lipid-to-peptide ratios in the presence of PEN or PEN_CF_. This indicates that the localization of these two peptide conjugates within lipid bilayers, and hence the mechanism of binding, differs from that of the other conjugates. In literature, it is reported that dissociation of assembled lipid vesicles in a peptide-lipid vesicle sample mixture, can be explained by peptide depletion from the outer membrane surface as a result of translocation into the interior of the lipid vesicles^51^. Here, experimental indications show that the CF-labeled PEN differed from the other peptide conjugates in its effect on the lipid membrane. Although binding of PEN_CF_ to the membrane was strong, it was seemingly of irreversible nature, and no reproducibility could be obtained in ITC experiments. According to the DLS measurements (Supplementary Fig. S5), the vesicle assembly behavior was completely different upon titration with PEN_CF_ as compared to that of all the other fluorophore-PEN conjugates investigated, as massive vesicle assembly was observed in a broad range of lipid-to-peptide ratios. PEN_CF_ has previously been observed to exhibit pronounced membrane binding in Caco-2 cell monolayers in contrast to PEN conjugated to any of the other tested fluorophore moieties^23^, supporting the hypothesis of strong and irreversible membrane interaction.

Previous ITC studies^52,53^ with titration of charged lipid vesicles into a sample cell containing PEN showed a  similar interruption of the sigmoidal curve shape, as observed for PEN and PEN_RhB_ in the present study. In those studies, it was proposed that this burst in the differential heat could be interpreted as the adsorption threshold at which PEN begins to penetrate into the lipid bilayer of the vesicles, after which also binding to the inner leaflet is initiated. The order in which these events occur is likely to be regulated by the accumulation of peptide at the membrane surface as a consequence of electrostatic interactions. The resulting enrichment in peptide content near the surface facilitates the binding event, which in turn destabilizes the bilayer and thus allows for the peptide to translocate across the bilayer. This interpretation is in line with our interpretation of vesicle assembly and dissociation, and thereby supports the hypothesis that vesicle assembly affects the ITC data.

The titration profiles of lipid vesicles into solutions of PEN labeled with TAMRA, NBD, MCA and PBA all follow the typical sigmoidal binding curve (Fig. 4). However, the enthalpy of peptide-membrane interaction for these four conjugates varies (average values): −0.38, −0.50, −0.85 and −1.5 kJ/mol, respectively (Table 1). This ranking does not correlate to the relative hydrophobicity of the labeled peptide conjugates^23^, and thus suggests that the enthalpy effect does not solely stem from hydrophobic interactions that otherwise could explain the negative binding enthalpy of immersion of the hydrophobic fluorophore moieties into the bilayer.

Evaluation of the number of lipid molecules surrounding each peptide molecule indicates that PEN occupies a larger surface area on the membrane as compared to the fluorophore-labeled peptide conjugates. Considering the size of the conjugated fluorophores, this is counter-intuitive. However, it correlates well with the obtained NR data (Fig. 6 and Supplementary Fig. S4), which suggest that the peptide conjugates, in contrast to native PEN, insert deep into the core of the lipid bilayer. This deep penetration into the lipid bilayer will leave a shorter peptide stretch to interact with the head groups of the lipids, thus covering less of the outer surface area of the lipid membrane. In addition, the higher stoichiometry of PEN (Table 1) might arise from a higher surface density of this peptide as compared to that of the labeled peptide conjugates.

### The hydrophobicity of the fluorophore labels determines the degree of membrane disturbance, perturbation, and penetration depth

The degree of membrane disturbance can be deduced from the QCM-D experiments (Fig. 5). SLBs are thin and rigid layers that are well coupled to the sensor surface; so increased dissipation (values exceeding ∼1 × 10^−6^) and/or overtone spreading are indications of disturbance of the rigid character of the SLBs. The conversion into a less rigid bilayer might arise from either peptide insertion into the SLB that disrupts the aligned structure of the SLB and/or from the incorporation of water into the SLB as a consequence of lipid removal from the SLB upon exposure to the peptide. The peak in the QCM-D frequency signal observed for both PEN_RhB_ and PEN_PBA_ (Fig. 5) indicates that lipid material was removed after exposure to these peptide conjugates, as this specific signal is a fingerprint for micellization of parts of the membrane^38,39^.

A process involving lipid removal is in agreement with the NR data for all compounds except for native PEN (Fig. 6, Supplementary Fig. S4 and Table S1). Moreover, from the NR data, it is evident that the structural properties of the SLBs were clearly affected by the exposure to the  fluorophore-conjugated PEN as observed by changes in the thickness of both lipid head groups and tail regions. For PEN, primarily head group association was found without significant effect on the lipid core of the membrane. This is in accordance with several studies stating that non-labeled PEN does not penetrate deeply into lipid bilayers, and thus does not significantly affect the lipid core of the membrane^5,54,55^. Thus, native PEN localizes mainly at the water-lipid interface, explaining why PEN in the concentration range tested in the present study only promotes a minor permeation of calcein through the bilayers of the lipid vesicles (Fig. 3). In contrast, both PEN_RhB_ and PEN_PBA_ induce high levels of calcein release starting at P/L ratios of 5 and 25, respectively, corresponding to a CPP concentration in the low micro-molar range (1 and 5 µM, respectively). This is consistent with the incorporation of these conjugates in the lipid core of the SLBs consistent with an increased membrane fluidization.

Major differences were observed regarding the extent of membrane disturbance induced by the various fluorophore-PEN conjugates as reflected by the differences in the degree of penetration into the hydrophobic lipid core of the bilayer. Naturally, the attachment of any hydrophobic fluorescent moiety to PEN will to some extent increase the hydrophobicity of the resulting conjugate. The hydrophobicity of PEN and the fluorophore-PEN conjugates relative to each other was previously assessed by measuring their retention to the stationary phase in RP-HPLC^23^. The relative hydrophobicity was found to be in the following order: PEN_PBA_ > PEN_RhB_ >> PEN_TAMRA_ > PEN_CF_ > PEN_MCA_ > PEN_NBD_ > PEN. Conjugates PEN_PBA_ and PEN_RhB_ exhibit significantly higher degrees of hydrophobicity as compared to the other conjugates, which do not differ considerably from each other. Indeed, PEN_PBA_ and PEN_RhB_ are the compounds that induce most drastic changes in the lipid membrane structure, as assessed by the various applied techniques in the present study. These two conjugates exert comparable effects on the lipid bilayer models with respect to induction of membrane permeabilization (Fig. 3), membrane disturbance (Fig. 5), and penetration depth into the lipid bilayer (Fig. 6 and Supplementary Fig. S4). Interestingly, these similarities in membrane effects occur despite the structural molecular differences between the two fluorophores.

Comparable results for effects on the planar SLBs were found for PEN_CF_ and PEN_TAMRA_ displaying structurally similar fluorophores, but carrying opposite charge at pH 7.4. According to the changes in frequency in the QCM-D experiments (Fig. 5), the amount of peptide adsorbed to the SLB is similar, and the NR profiles resemble each other in such a way that the level of disturbance of the bilayers is comparable (Fig. 6). For PEN_NBD_, it is not clear from QCM-D whether lipid molecules are removed from the SLB upon exposure to the conjugate. Nonetheless, lipid removal must have occurred, since membrane thinning was necessary in order to obtain an appropriate model of the NR data for PEN_NBD_. However, in this fit, no significant amount of peptide seemed to be present in the lipid tail region of the remaining SLB.

In our recent report on fluorophore-dependent membrane effect^23^, the extent of cellular membrane translocation, cytotoxic effects and induction of morphological changes applying a variety of cell lines were investigated. Specifically, the use of PBA and RhB as labels was found to be problematic in the assessment of membrane permeation propensity of PEN. PEN conjugated with these two fluorophores induced considerable cytotoxic effects to the tested cells at surprisingly low peptide concentrations, which could result from increased translocation of the conjugate across the cell membrane as compared to native PEN. Despite the high complexity of the cell membrane of living cells as compared to the simple phospholipid bilayers used in the present study, the *in vitro* findings correlate well with our observations, and therefore use of simple membrane models and advanced complementary surface-sensitive techniques is justified as a predictive tool for peptide-membrane interactions.

## Conclusion

With the results presented in this study, we emphasize that caution has to be taken when applying fluorophore labeling of peptides for biomembrane-interaction studies. The membrane disturbance was found to be highly fluorophore-dependent with the most pronounced effects observed for the two most hydrophobic labels, namely RhB and PBA. Upon labeling of PEN with these fluorophores, the resulting conjugates were found to localize in the lipid core of SLBs. This resulted in increased membrane permeabilization and lipid removal, which was in contrast to the effect of native PEN. These two fluorophore-PEN conjugates induced significant structural changes in the membrane, and as all the other conjugates they displayed a membrane-thinning effect. Interestingly, the use of high-resolution NR revealed that PEN conjugated to the fluorophores CF, TAMRA, and NBD also induced substantial effects to the bilayer. The present work shows that application of complementary biophysical and surface-sensitive techniques can be used with advantage to assess the possible impact that even small structural modifications can have on biomembrane interaction.

## Electronic supplementary material

Supplementary information
