# Supplementary material for: Fluorophore labeling of a cell-penetrating peptide significantly alters the mode and degree of biomembrane interaction
Source: Sci Rep. 2018 Apr 20;8:6327. doi: 10.1038/s41598-018-24154-z (PMC5910404; doi:10.1038/s41598-018-24154-z)
Supplement: Supplementary file 1 — Supplementary information [file 41598_2018_24154_MOESM1_ESM.docx]

**Supplementary information**

**Fluorophore labeling of a cell-penetrating peptide significantly alters the mode and degree of biomembrane interaction**

Sofie Fogh Hedegaard, Mohammed Sobhi Derbas, Tania Kjellerup Lind, Marina Robertnova Kasimova, Malene Vinther Christensen, Maria Høtoft Michaelsen, Richard Campbell, Lene Jorgensen, Henrik Franzyk, Marité Cárdenas, Hanne Mørck Nielsen

**Quartz crystal microbalance with dissipation monitoring (QCM-D)**


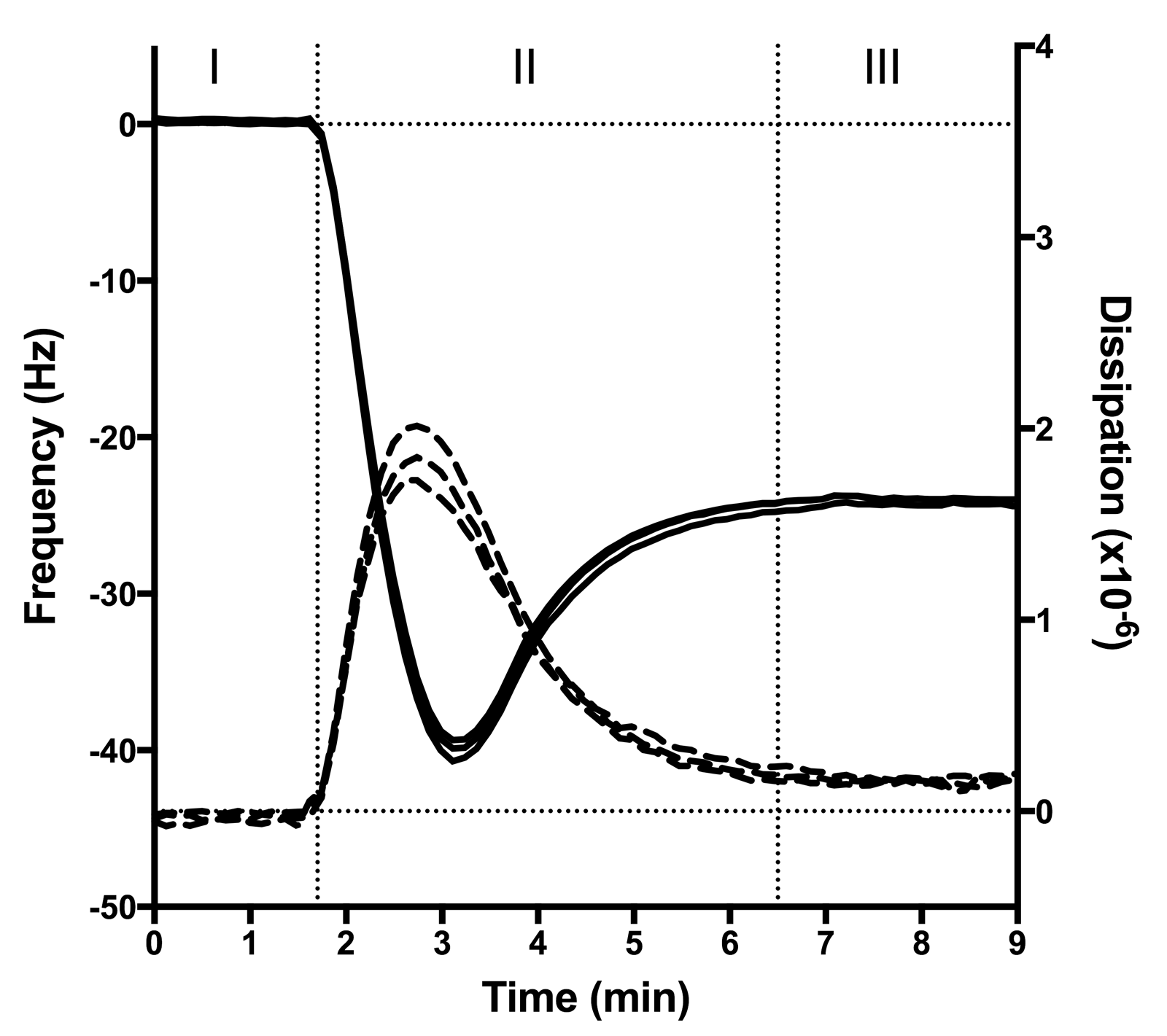


Figure S1: Typical QCM-D traces of SLB formation by vesicle fusion from 0.1 mg/mL lipid suspension composed of POPC:POPG (80:20 mol%) in 2 mM CaCl_2_ at 25 °C. Three overtones; 5^th^, 7^th^ and 9^th^ are shown. The sections represent: I) A clean crystal surface in 2 mM CaCl_2_, II) surface adsorption of lipid vesicles in 2 mM CaCl_2_ followed by vesicle rupture, which leads to III) a stable SLB in 2 mM CaCl_2_.

**Neutron reflection**


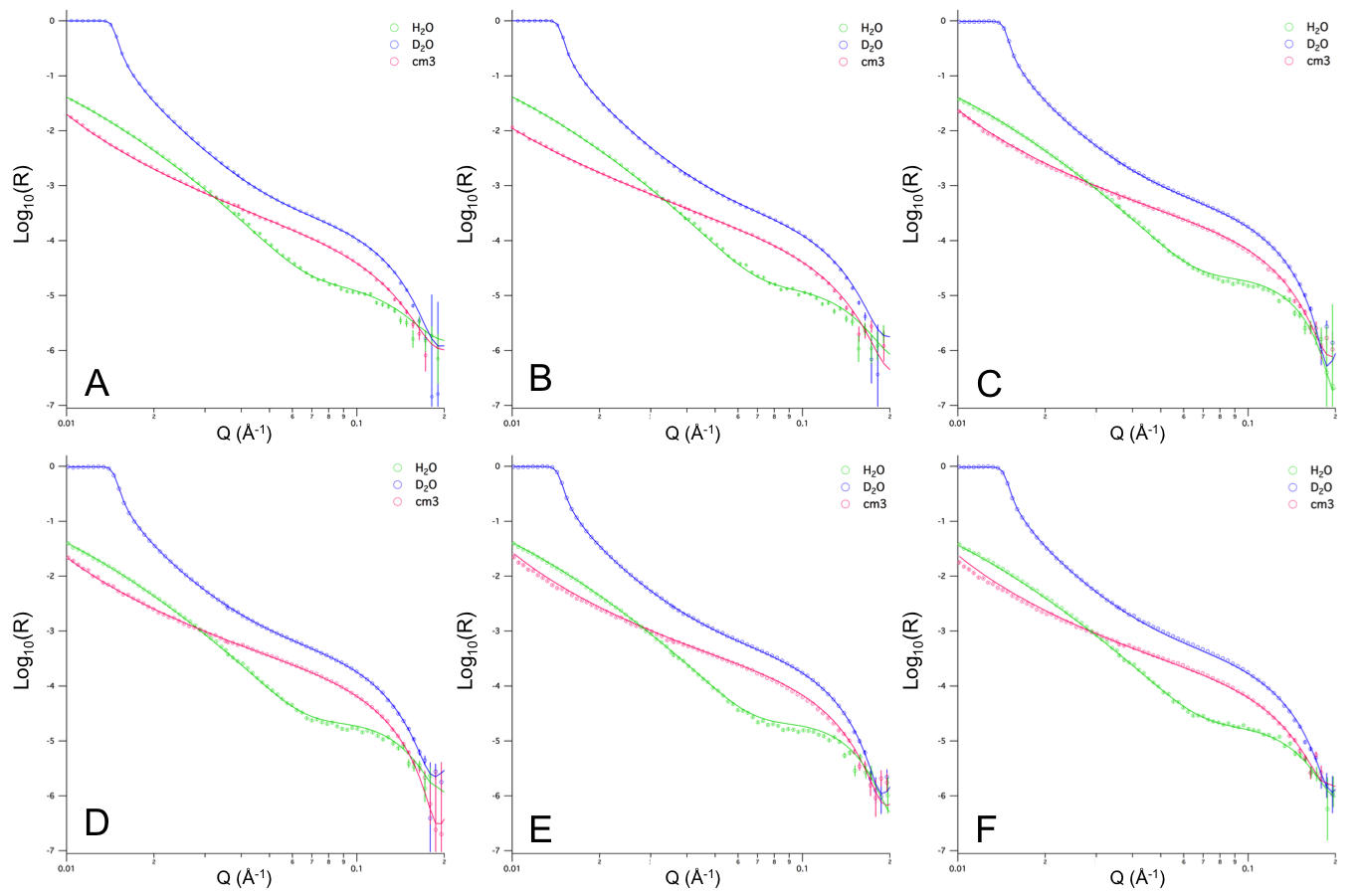


Figure S2: Neutron reflectivity profiles of POPC:POPG (80:20 mol%) SLBs measured in three isotopic contrasts: blue: D_2_O, pink: cm3, and green: H_2_O. The solid lines represent the best fit to the data. The following conjugates were applied to the specific SLBs: A: PEN, B: PEN_RhB_, C: PEN_PBA_, D: PEN_NBD_ E: PEN_CF_, F: PEN_TAMRA_.


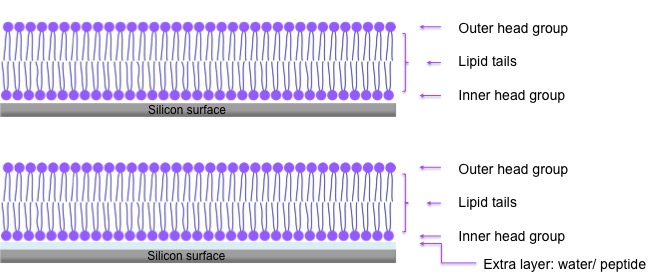


Figure S3: Simple illustration of the three- and four-layer models as applied in the fitting of most of the NR data presented in Table S1. Neutrons are reflected from below the solid surface and reflect at the liquid-solid interface. It is the interference pattern between the reflected and refracted beams at the various layers with distinct isotopic composition that give rise to the reflectivity pattern observed in the profiles shown in Figure 6, Figure S2 and S4.

Table S1: NR fitted parameters for penetratin (PEN)

| Bilayer | Thickness (Å) | Dry coverage (%) | Roughness (Å) |
| --- | --- | --- | --- |
| Inner head group | 7.5 ± 1 | 58 ± 1 | 2 ± 0.4 |
| Lipid tails | 29.5 ± 1 | 88 ± 1 | 5 ± 0.1 |
| Outer head group | 7.5 ± 1 | 58 ± 1 | 5 ± 0.1 |

| + PEN | Thickness (Å) | Dry coverage (%) | Roughness (Å) |
| --- | --- | --- | --- |
| Inner head group | 11.0 ± 1 | 44 ± 1 | 4± 0.1 |
| Lipid tails | 30.1 ± 1 | 99 ± 1 | 4 ± 0.1 |
| Outer head group | 10.0 ± 1 | 34 ± 1 | 5 ± 0.1 |

Table S2: NR fitted parameters for PEN_RhB_

| Bilayer | Thickness (Å) | Dry coverage (%) | Roughness (Å) |
| --- | --- | --- | --- |
| Inner head group | 8.0 ± 1 | 56 ± 1 | 2 ± 0.3 |
| Lipid tails | 29.0 ± 1 | 94 ± 1 | 4 ± 0.2 |
| Outer head group | 8.0 ± 1 | 56 ± 1 | 5 ± 0.1 |

| + PEN-RhB | Thickness (Å) | Dry coverage (%) | Roughness (Å) |
| --- | --- | --- | --- |
| Peptide layer | 4.4 ± 1 | 16 ± 1 | 5 ± 0.2 |
| Inner head group | 9.0 ± 1 | 52 ± 1 | 2 ± 0.5 |
| Lipid tails | 24.0 ± 1 | 100 ± 1 | 6 ± 0.2 |
| Outer head group | 6.8 ± 1 | 37 ± 1 | 6 ± 0.1 |

Table S3: NR fitted parameters for PEN_NBD_

| Bilayer | Thickness (Å) | Dry coverage (%) | Roughness (Å) |
| --- | --- | --- | --- |
| Inner head group | 7.0 ± 1 | 70 ± 1 | 2 ± 0.1 |
| Lipid tails | 30.2 ± 1 | 100 ± 1 | 2 ± 0.1 |
| Outer head group | 7.4 ± 1 | 56 ± 1 | 5 ± 0.1 |

| + PEN-NBD | Thickness (Å) | Dry coverage (%) | Roughness (Å) |
| --- | --- | --- | --- |
| Water layer | 4.0 ± 1 | 0 ± 1 | 3 ± 0.5 |
| Inner head group | 11.2 ± 1 | 77 ± 1 | 2 ± 0.1 |
| Lipid tails | 26.0 ± 1 | 100 ± 1 | 1 ± 0.1 |
| Outer head group | 5.8 ± 1 | 80 ± 1 | 2 ± 0.1 |
| Water layer | 29.3 ± 1 | 0 ± 1 | 1 ± 0.2 |
| Mixed lipid/peptide | 18.6 ± 1 | 11 ± 1 | 5 ± 0.6 |

Table S4: NR fitted parameters for PEN_PBA_

| Bilayer | Thickness (Å) | Dry coverage (%) | Roughness (Å) |
| --- | --- | --- | --- |
| Inner head group | 7.1 ± 1 | 73 ± 1 | 2 ± 0.1 |
| Lipid tails | 29.8 ± 1 | 100 ± 1 | 2 ± 0.1 |
| Outer head group | 8.6 ± 1 | 60 ± 1 | 2 ± 0.1 |

| + PEN-PBA | Thickness (Å) | Dry coverage (%) | Roughness (Å) |
| --- | --- | --- | --- |
| Peptide layer | 4.0 ± 1 | 31 ± 1 | 2 ± 0.1 |
| Inner head group | 10.0 ± 1 | 70 ± 1 | 5 ± 0.1 |
| Lipid tails | 26.5 ± 1 | 100 ± 1 | 2 ± 0.1 |
| Outer head group | 7.0 ± 1 | 48 ± 1 | 2 ± 0.1 |


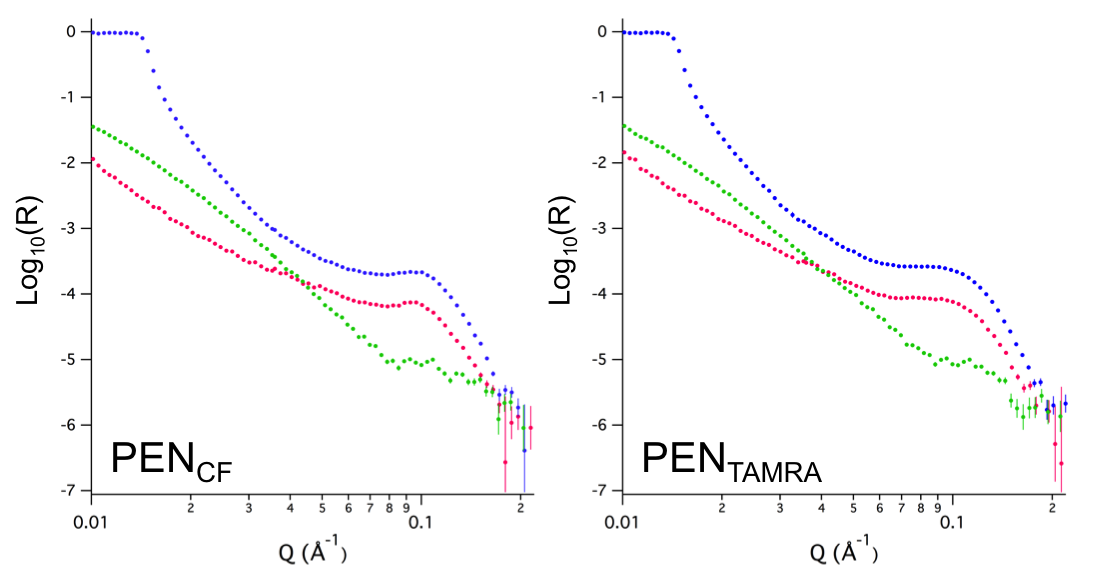


Figure S4: Neutron reflectivity profiles of POPC:POPG (80:20 mol%) SLBs after interaction with PEN_CF_ or PEN_TAMRA_. The CPPs were added in a concentration of 5 µM in HEPES buffer, pH 7.4. The SLBs were measured in three isotopic contrasts; blue: D_2_O, pink: cm3, and green: H_2_O. The inner head groups are in contact with the silicon surface and the outer head group faces the bulk solution.

**Dynamic light scattering**

DLS titration experiments were carried out by manual titration of 4 mM liposomes into a 70 µM 10 mM HEPES peptide solution (40 µM for PEN). The mixture was vortexed briefly in the cuvette and the size distribution was measured immediately thereafter. Freshly prepared liposomes of the same batch were used for all peptide samples. A Nano-ZS Zetasizer ZEN3600 from Malvern Instruments (Worcestershire, UK) equipped with a 4 mW He–Ne laser operating at λ_0_= 633 nm and 173° detection optics was used for the measurements. Each value reported is the average of three consecutive measurements.


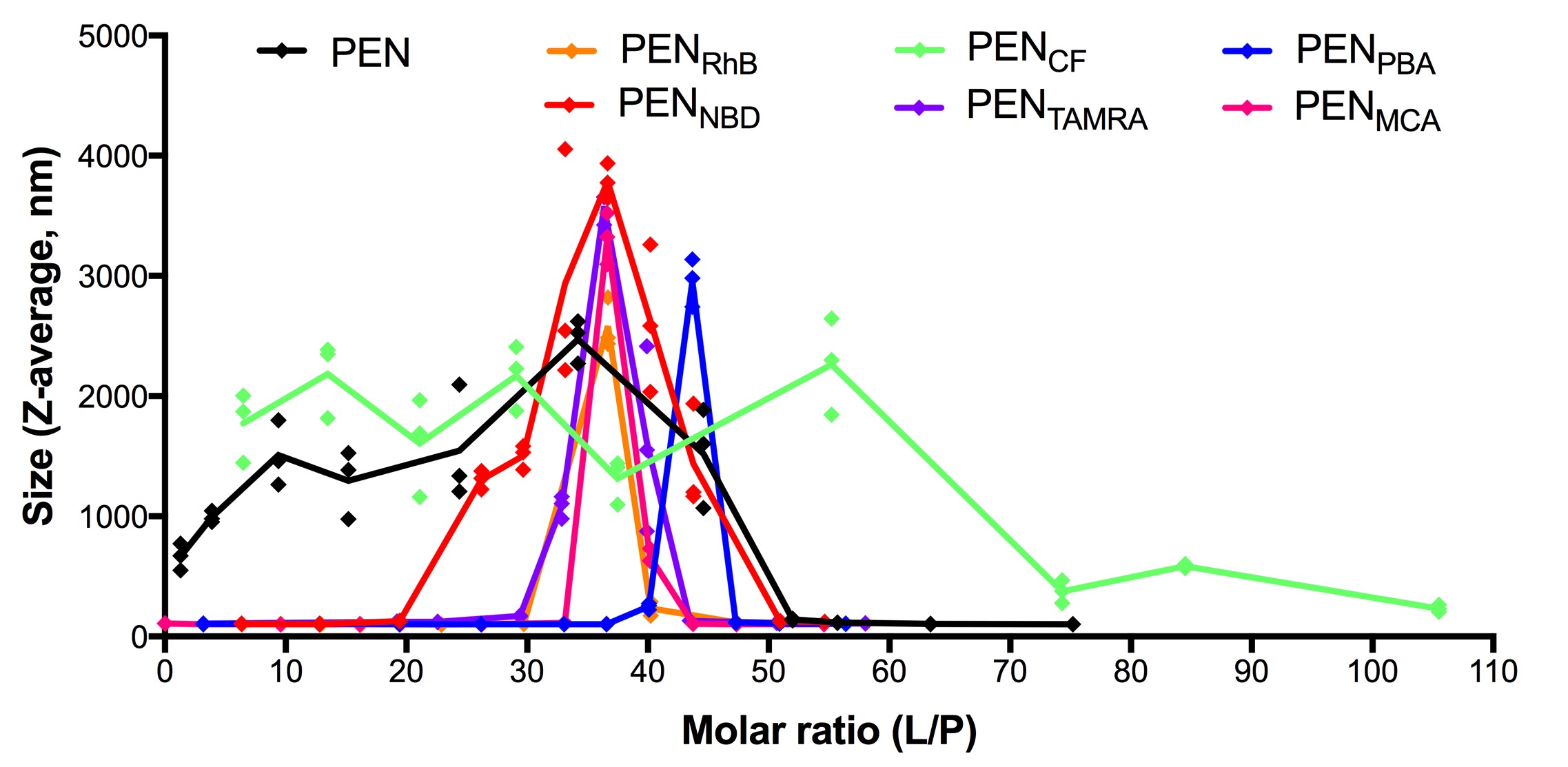


Figure S5: Dynamic light scattering results. Representative intensity distributions of the diameter of 5 mM POPC:POPG (80:20 mol%) lipid vesicles upon titration into 70 µM peptide (40 µM for PEN) buffer solution pH 7.4. The initial size of the lipid vesicles was approximately 100 nm in diameter with a polydispersity index (PDI) of <0.15. Prior to and after the obvious vesicle assembly, the vesicles were approximately 100 nm. The symbols represent technical replicates and the connecting lines are mean values for clarity.
